# Postprandial Effects on ENaC-Mediated Sodium Absorption

**DOI:** 10.1038/s41598-019-40639-x

**Published:** 2019-03-12

**Authors:** Gregory Blass, Christine A. Klemens, Michael W. Brands, Oleg Palygin, Alexander Staruschenko

**Affiliations:** 1 Department of Physiology, Medical College of Wisconsin, Milwaukee, WI 53226 USA; 2 Cardiovascular Center, Medical College of Wisconsin, Milwaukee, WI 53226 USA; 3 Clement J. Zablocki VA Medical Center, Milwaukee, WI 53295 USA; 4 Department of Physiology, Medical College of Georgia, Augusta University, Augusta, GA 30901 USA; 5 Present Address: Western Kentucky University, Bowling Green, KY 42101 USA

## Abstract

Recent studies have suggested that postprandial increases in insulin directly contribute to reduced urinary sodium excretion. An abundance of research supports the ability of insulin to augment epithelial sodium channel (ENaC) transport. This study hypothesized that ENaC contributes to the increase in renal sodium reabsorption following a meal. To test this, we used fasted or 4 hour postprandial Sprague Dawley rats to analyze ENaC expression and activity. We also assessed total expression of additional sodium transporters (Na^+^-Cl^−^ cotransporter (NCC), Na^+^-K^+^-2Cl^−^ cotransporter (NKCC2), and Na^+^-K^+^-ATPase (NKA)) and circulating hormones involved in the renin-angiotensin-aldosterone system (RAAS). We found that after carbohydrate stimulus, ENaC open probability increased in split-open isolated collecting duct tubules, while ENaC protein levels remained unchanged. This was supported by a lack of change in phosphorylated Nedd4-2, an E3 ubiquitin ligase protein which regulates the number of ENaCs at the plasma membrane. Additionally, we found no differences in total expression of NCC, NKCC2, or NKA in the postprandial rats. Lastly, there were no significant changes in RAAS signaling between the stimulated and fasted rats, suggesting that acute hyperinsulinemia increases ENaC activity independent of the RAAS signaling cascade. These results demonstrate that insulin regulation of ENaC is a potential mechanism to preserve sodium and volume loss following a meal, and that this regulation is distinct from classical ENaC regulation by RAAS.

## Introduction

Many diabetic studies in the past have focused on physiological changes that occur during fasting; however, the importance of postprandial metabolic effects, particularly the role of systemic insulin signaling, is becoming increasingly realized. Fluctuating insulin levels resulting from increases in circulating glucose represent a normal aspect of our metabolic regulation; however, most previous studies have utilized all-or-none insulin replacement strategies similar to the pathologies seen in type 1 diabetes. Evolutionarily, since mammals are designed to conserve sodium due to our prehistoric diet, it would make sense that renal insulin helps prevent sodium excretion following an osmotic load from a meal. The effect of insulin stimulation on sodium reabsorption in epithelia and in the kidney and on a number of specific channels and transporters is well known, and has a long historic precedence^1,2^. It has been reported that insulin can increase Na^+^-HCO_3_^−^ cotransporter (NBCe1) activity in the proximal tubule^3^, Na^+^-K^+^-2Cl^−^ cotransporter (NKCC2) expression in the thick ascending limb^4^, enhance phosphorylation of the Na^+^-Cl^−^ cotransporter (NCC) in the distal convoluted tubule^5,6^, and increase of Na^+^-K^+^-ATPase (NKA) activity in the collecting duct^7^. The role of insulin in control of sodium-dependent glucose transporters (SGLT), especially SGLT2, is also established^8,9^. This is particularly important since SGLT2 inhibitors have recently been used as drugs to treat type 2 diabetes^10,11^.

In the kidney, epithelial sodium channels (ENaC) are located on the apical membrane of principal cells in the aldosterone-sensitive distal nephron where they are tightly controlled by various hormones and mediate fine-tuning of sodium absorption in the kidney^12^. We and others have shown previously that insulin augments ENaC expression and activity^13–20^. As an example, single-channel analysis in freshly isolated split-open tubules demonstrated that the ENaC activity was acutely activated by insulin, and insulin receptor knock out mice have significantly lower activity compared to their wild-type littermates^16^.

Recent studies by Irsik, *et al*.^21,22^ have utilized a sophisticated insulin-clamping technique in rats, which allowed them to test the role of daily variations in insulin on sodium excretion. They found that rats whose insulin was clamped and prevented from increasing in response to a carbohydrate supplement increased sodium excretion over the first 4 hours post carbohydrate administration^22^. This suggests that the postprandial rise in circulating insulin is necessary to prevent excessive renal volume and sodium losses^21^ and implicates insulin as part of a metabolic control system regulating corporal sodium homeostasis. Establishing insulin as a normal regulatory factor opens new avenues of investigation to determine which transporters may be affected within this short postprandial time frame.

As described above, multiple studies have demonstrated the ability of insulin to increase sodium transport via ENaC, and while pathologically high levels of circulating insulin are hypothesized to help contribute to the development of hypertension, the role of naturally fluctuating levels of insulin on ENaC under normal physiological conditions have yet to be investigated, particularly in the postprandial time frame. In this study, we investigated the hypothesis that ENaC activity would increase during the postprandial period, likely in response to temporary hyperinsulinemia, and that this increase in ENaC activity could be essential to prevent postprandial sodium loss. To test this hypothesis, we recorded ENaC from isolated split-open cortical collecting ducts and measured its activity. Western blotting was further utilized to measure expression levels of ENaC subunits and other sodium transporters such as NCC, NKCC2, and αNKA, as well as the ENaC-targeting E3 ubiquitin ligase Nedd4-2. We also investigated if there were any changes to the renin angiotensin aldosterone (RAAS) system to determine whether the insulin signaling was working independently on ENaC or part of a more complex, intertwined regulatory control system.

## Results

### Blood glucose, electrolytes and insulin levels

**Figure 1:**
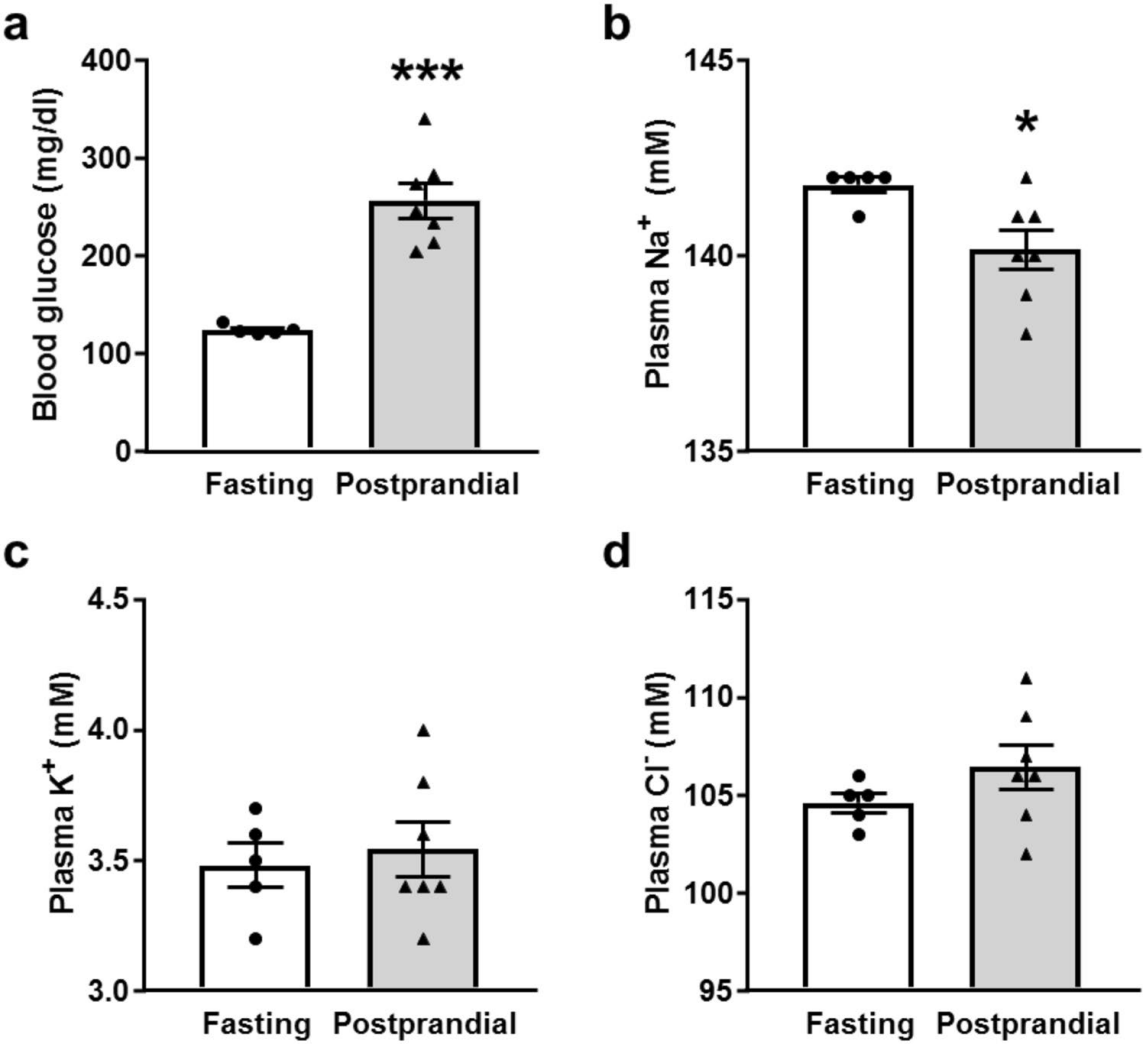
Blood glucose and plasma electrolytes following the meal stimulus. Summary graphs of (**a**) blood glucose, (**b**) plasma Na^+^, (**c**) K^+^, and (**d**) Cl^−^ concentrations in fasted and postprandial Sprague-Dawley rats. *p < 0.05, ***p < 0.001, respectively, N ≥ 5 rats.

**Figure 2:**
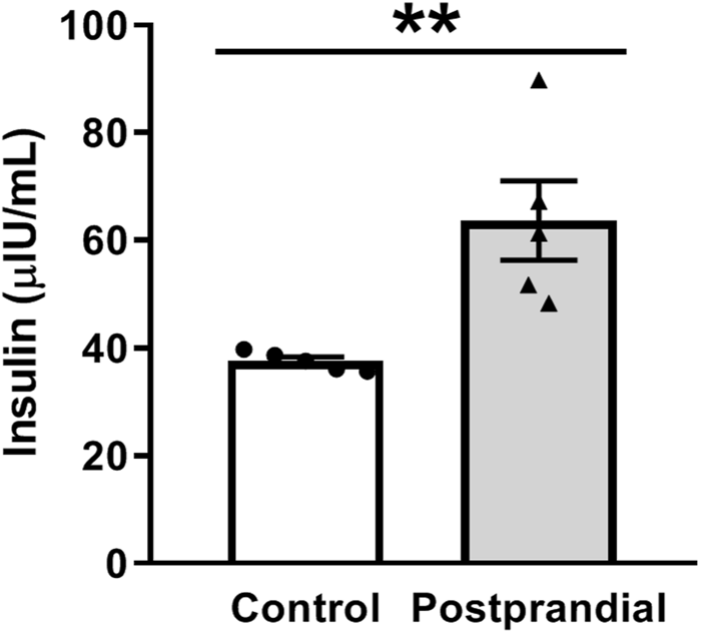
Plasma insulin levels from fasted or postprandial rats. Relative serum insulin levels as determined by ELISA. The insulin levels of the postprandial rats are significantly increased compared to controls 4 hours after the meal stimulus. N = 5 rats, **p < 0.01.

In order to test the postprandial renal response, male Sprague Dawley rats were fasted overnight and some were given a 10–11 g portion of Supplical gel to simulate rapid consumption and the glucose and insulin response following a single meal. After 4 hours, the control and postprandial rats were euthanized and blood and kidney tissues were collected and used for corresponding measurements. As expected, blood glucose levels were significantly elevated in the postprandial group (Fig. 1a). Electrolyte analysis revealed that plasma sodium concentrations were slightly but significantly reduced, while plasma K^+^ and Cl^−^ concentrations did not change in the postprandial group relative to the control rats (Fig. 1b–d). Additionally, we tested plasma insulin levels in the postprandial group, and found that the insulin was still significantly elevated 4 hours following the meal supplement compared to control (Fig. 2).

### ENaC activity and expression in postprandial rats

**Figure 3:**
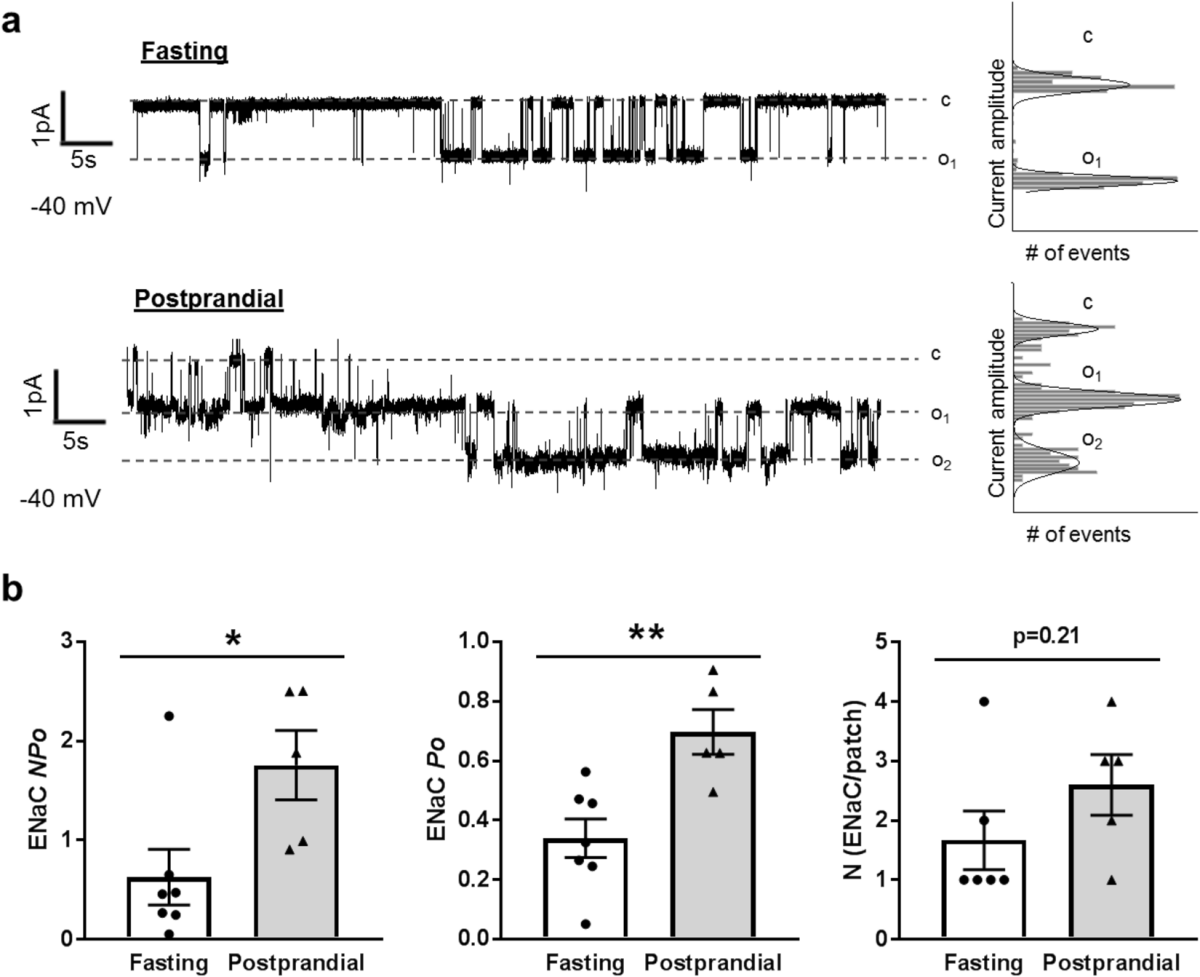
ENaC activity in isolated cortical collecting duct (CCD) tubules. (**a**) Representative current traces from cell-attached patches containing ENaC recorded from the apical membrane of split-open CCD cells of fasted or postprandial Sprague-Dawley rats. Event probability histograms of the representative traces showing the distribution of open and closed states are depicted to the right of the trace. (**b**) Summary graphs of ENaC activity (*NP*_*o*_), channel open probability (*P*_*o*_) and the average number of channels per patch from isolated CCDs. *p < 0.05, **p < 0.01, respectively, N ≥ 5 rats.

**Figure 4:**
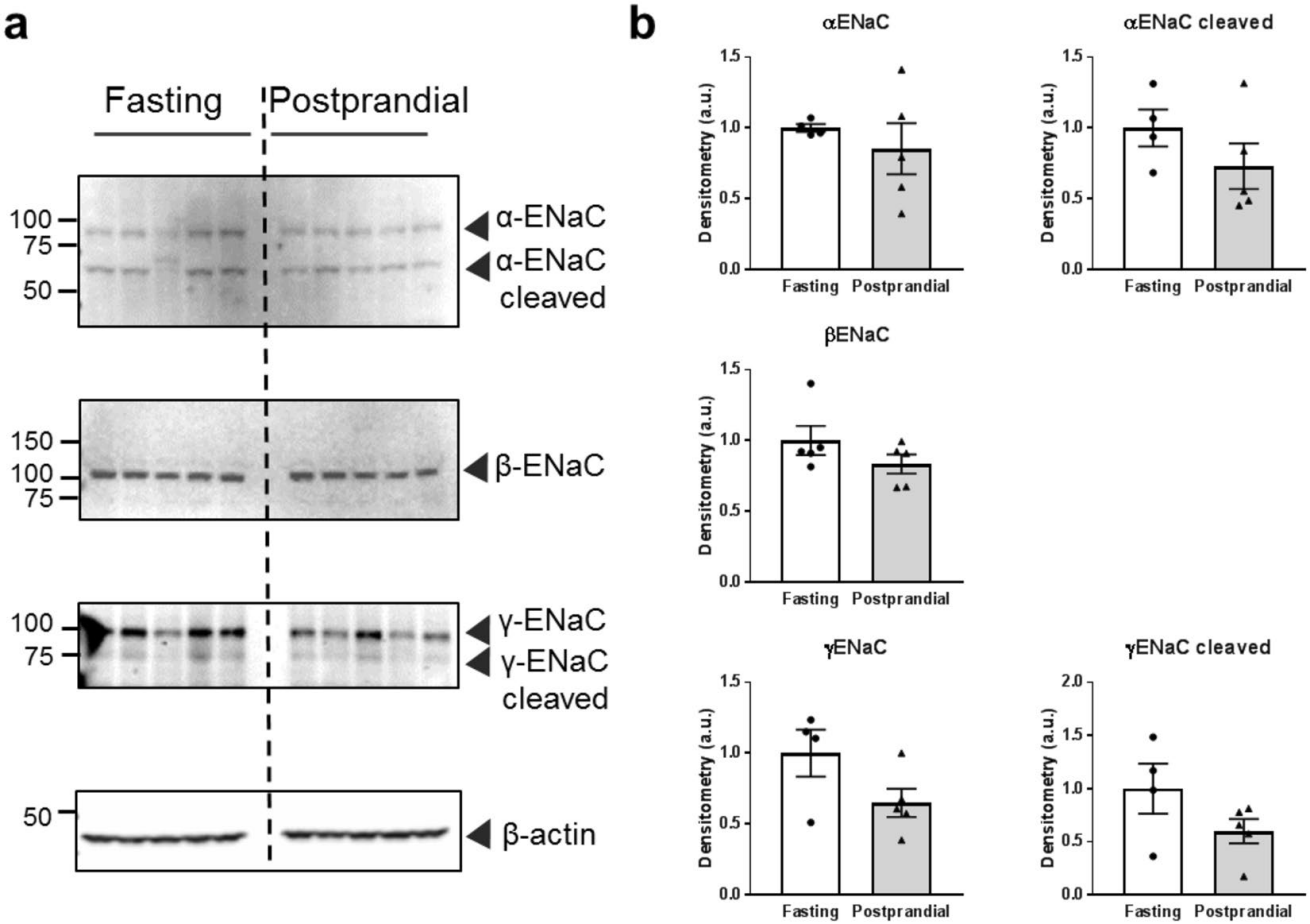
Expression of ENaC subunits in fasted or postprandial rats. (**a**) Western blots of α-, β-, and γ-ENaC subunit expression. (**b**) Summary graphs of ENaC subunit expression after normalization to β-actin loading control. Each lane represents 1 rat, N = 5 rats in each group.

Numerous *ex vivo* and *in vitro* studies have shown that ENaC activity increases following insulin stimulation^13,16–20,23–26^. Tiwari *et al*.^17^ found that reduced urinary sodium excretion following a meal in mice could be reversed by pretreatment with the ENaC antagonist benzamil, suggesting ENaC plays a key role in insulin-regulated sodium reabsorption. To date, however, postprandial ENaC activity has not been examined. To test whether ENaC activity increases following a meal, we made single channel recordings of ENaC activity in isolated split-open collecting duct tubules from fasted or postprandial rats. Representative patch-clamp traces are shown in Fig. 3a. Patch-clamp measurements demonstrated a significant increase in ENaC activity in the tubules from postprandial rats. This increase in activity resulted from an increase in channel open probability (*P*_*o*_) rather than a change in the number of channels at the surface (Fig. 3b). These results are consistent with previous research that found insulin increased ENaC *P*_*o*_ in WT mouse collecting duct cells, but not in collecting duct cells lacking an insulin receptor^15,16^. Additionally, we examined ENaC subunit expression levels via Western blot, as some previous literature suggests that insulin-mediated regulation can alter subunit expression. We found no significant expression level changes in α-ENaC (full length or cleaved) or β-ENaC consistent with earlier studies^17^. The full-length and cleaved γ-ENaC trended towards a decrease, but was not significantly different (Fig. 4). These results demonstrate that ENaC activity increases following meal consumption through an increase in *P*_*o*_, and further support the role of ENaC in increased postprandial sodium reabsorption.

### Postprandial Nedd4-2 expression

**Figure 5:**
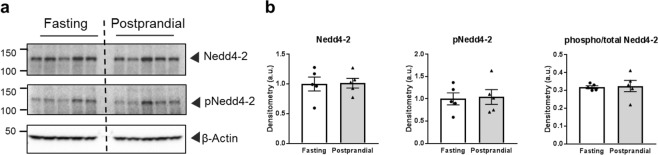
Total and phosphorylated Nedd4-2 expression in fasted or postprandial rats. (**a**) Western blots of Nedd4-2 and phosphorylated Nedd4-2 (pNedd4-2). (**b**) Summary graphs of band densitometry after normalization to β-actin loading control, and also the ratio of phosphorylated to total Nedd4-2. Each lane represents 1 rat, N = 5 rats in each group.

In collecting duct cells, the serum and glucocorticoid-regulated kinase (SGK1) is an important nexus of ENaC regulation through a variety of hormonal stimulation pathways including insulin^14,18,19,23,25,27–29^. SGK1 activity alters the amount of surface ENaC by phosphorylating the E3 ubiquitin ligase, Nedd4-2^30–32^. Nedd4-2 binding to ENaC reduces the number of ENaCs in the plasma membrane by targeting the channels for endosomal internalization, and phosphorylation of Nedd4-2 by SGK1 prevents Nedd4-2 from binding to ENaC, thereby reducing internalization and increasing the number of channels at the plasma membrane^30–32^. During insulin signaling, activation of the phosphatidyl inositol 3-kinase (PI3K) signaling transduction cascade results in changes to SGK1 which increase Na^+^ reabsorption via ENaC, although there is some debate within the field as to whether the insulin stimulation increases SGK1 expression, phosphorylation to activate SGK1 or a combination of both^14,17,18,20,23,25,29^. To bypass the potential variability of SGK1 expression or activation, we interrogated the downstream effect of SGK1 that directly impacts ENaC, namely Nedd4-2 phosphorylation at 4 hours. As demonstrated in Fig. 5, there is no significant difference in total or phosphorylated Nedd4-2 protein expression. While it is possible and likely that SGK1 is impacting ENaC at earlier time points in the insulin signaling cascade, at this time point we do not observe evidence of SGK1 activity on Nedd4-2. This is consistent with our electrophysiological data demonstrating that ENaC open probability but not surface channel number is affected in the 4 hour postprandial group.

### Distal tubule sodium transporter expression

**Figure 6:**
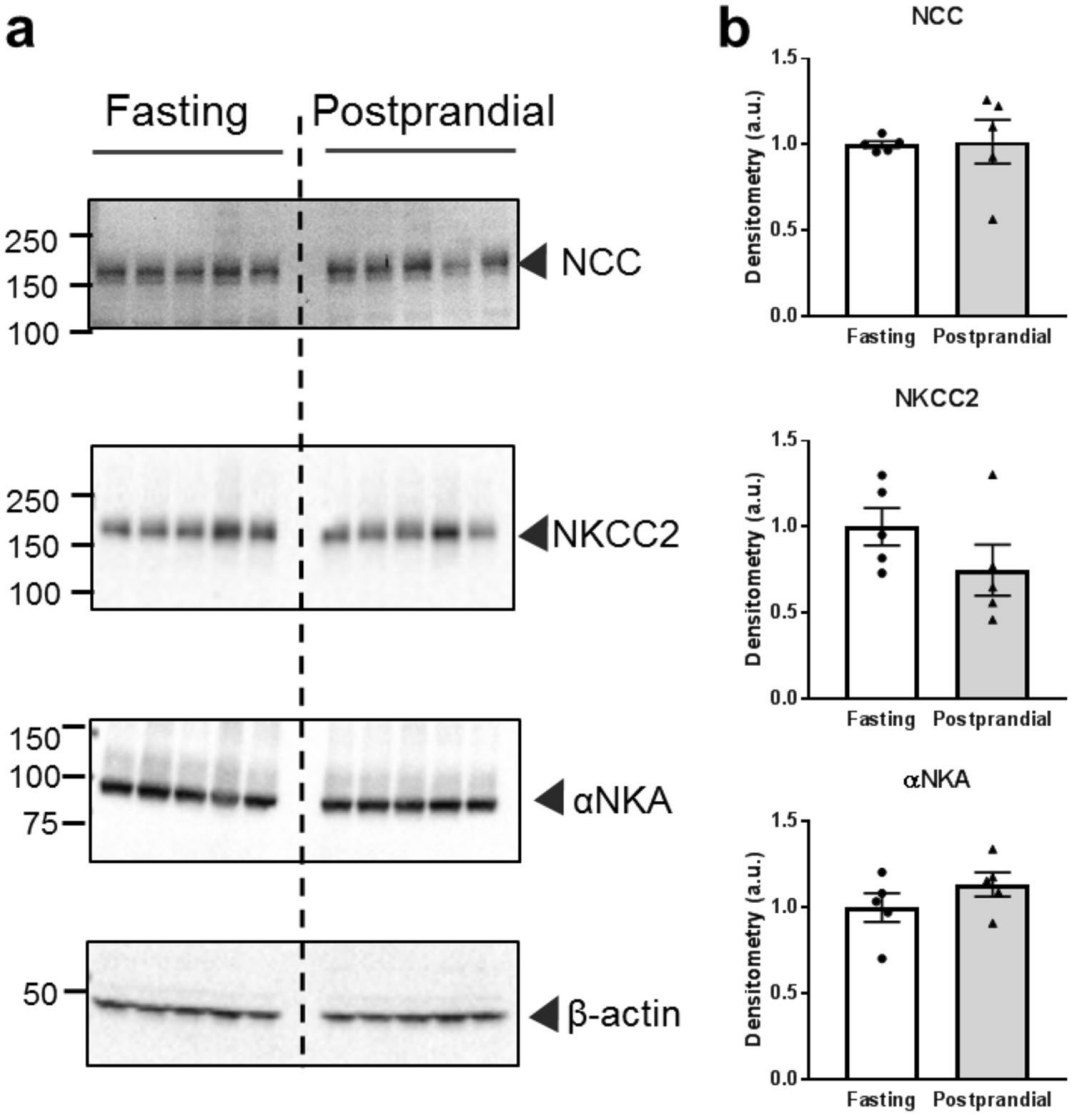
Expression of NCC, NKCC2 and αNKA in fasted or postprandial rats. (**a**) Western blots demonstrating NCC, NKCC2, and α-Na^+^/K^+^ ATPase (αNKA) expression. (**b**) Summary graphs of band densitometry after normalization to β-actin loading control. Each lane represents 1 rat, N = 5 rats in each group.

In addition to ENaC, several other Na^+^ transporters in the distal nephron are sensitive to insulin signaling, including NCC^5,33,34^, NKCC^4^, and NKA^7^. In our experimental model, 4 hours following a meal stimulus, we did not detect any significant changes to total NCC, NKCC2, or αNKA protein expression relative to controls (Fig. 6). While we did not observe any expression changes, this does not exclude the possibility that there were activity changes to these transporters and further studies are needed to assess this potential effect.

### Analysis of circulating Renin-Angiotensin-Aldosterone-System (RAAS) molecules

**Figure 7:**
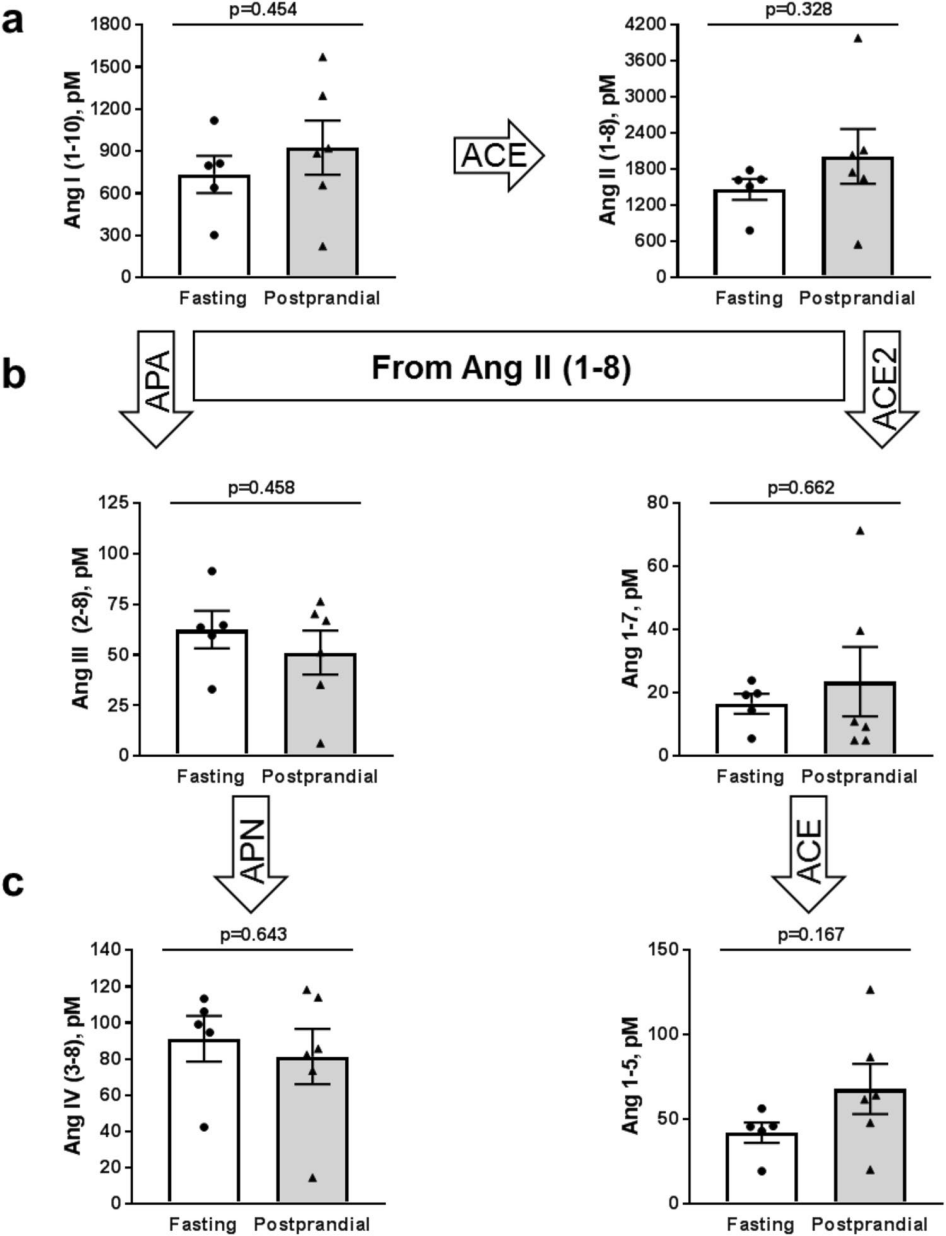
Angiotensin peptide plasma expression levels. Equilibrium levels of (**a**) Ang I (1–10) and Ang II (1–8), (**b**) And III (2–8), and Ang-(1–7), and (**c**) Ang IV (3–8) and Ang 1–5. There were no significant differences between fasted and postprandial rats; *p-*values are given for each graph. ACE, angiotensin converting enzyme; APA, aminopeptidase A; APN, aminopeptidase N.

**Figure 8:**
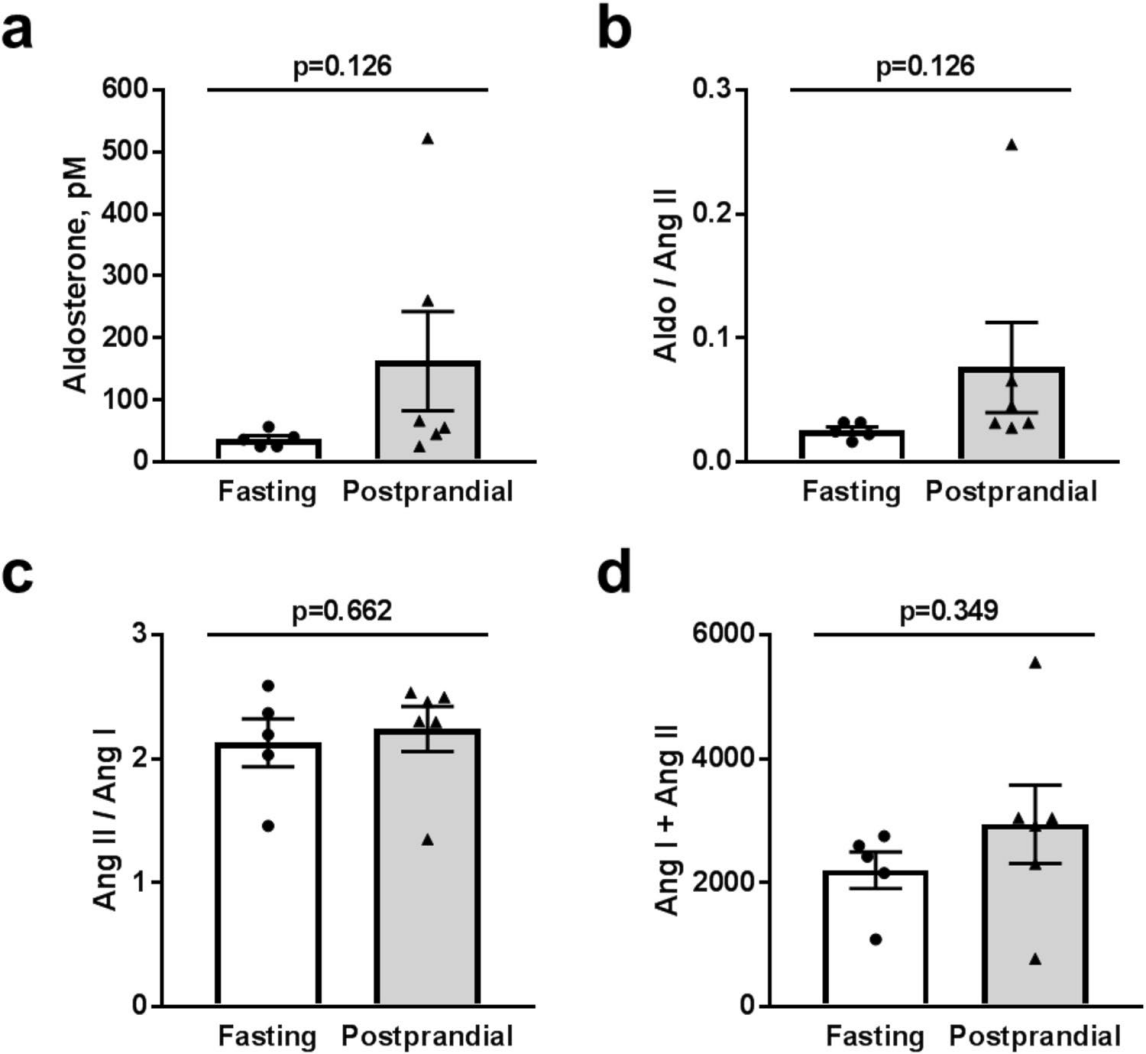
Aldosterone levels and ACE and renin activity. (**a**) Summary graph of aldosterone level in the fasting and postprandial rats. (**b**) Ratio of aldosterone and Ang II (AA2 ratio), representative of changes in the adrenal response to Ang II. (**c**) The ratio of Ang II/Ang I demonstrating angiotensin-converting enzyme (ACE) activity and (**d**) the sum of Ang I and Ang II giving circulating plasma renin activity (PRA). There were no significant differences between fasted and postprandial rats; *p* values are given for each graph.

RAAS is critically involved in the regulation of water and electrolyte balance and is responsible for the control of a variety of complex physiological functions, including ENaC-mediated sodium absorption^35,36^. We examined whether different RAAS components were modulated in acute postprandial experiments, and therefore involved in our observed changes in ENaC activity. We used mass spectrometry-based quantification of constituents of RAAS from Sprague Dawley rats after fasting or 4 hours post supplical consumption in equilibrated heparin plasma samples. These analyses included all angiotensin (Ang) derivatives, aldosterone levels, and ratios of different derivatives to give surrogate measurements of angiotensin-converting enzyme (ACE) activity (Ang II/Ang I), plasma renin activity (PRA, Ang I + Ang II), and the adrenal response to AngII (AA2-Ratio). We found that there were no significant changes in circulating Ang I or Ang II (Fig. 7a) in postprandial rats relative to fasted controls. Additionally, we examined the different derivatives of Ang II (Fig. 7b,c), but also found no significant differences between the two groups. Lastly, we analyzed plasma aldosterone levels, ACE and renin activity (as ratios of Ang II/Ang I and the sum of Ang I and Ang II, respectively (Fig. 8) and also did not observe any significant changes. While aldosterone does appear to trend upward in the postprandial group, the aldosterone levels (0.162 ± 80.05 nM) are still below the reported half maximal activation K_1/2_ (approximately 0.5 nM) for high-affinity aldosterone binding to the mineralocorticoid receptor in a mouse cortical collecting duct cell model^37^. These results suggest that postprandial increases in ENaC activity are independent from changes in RAAS signaling, further implicating ENaC as an important contributor to increased sodium retention immediately following a meal, likely through insulin stimulation.

## Discussion

Recent studies have hypothesized a critical role for the regular increases in insulin that occur throughout the day in association with meal-induced hyperglycemia and provided evidence for a powerful postprandial effect of insulin to conserve sodium after meals^21,22^. Rats that were prevented from raising circulating insulin levels above baseline had significant urinary sodium and volume losses over 24 hours of *ad libitum* eating and after a glucose bolus^21^. Urinary sodium excretion over 24 hours increased significantly in rats that could not increase circulating insulin above normal while they had *ad libitum* access to standard chow. The exaggerated natriuresis over 24 hours resulted from prevention of meal-induced hyperinsulinemia and the postprandial increase in insulin-mediated sodium conservation^21^. Further studies, utilizing a novel approach for renal-artery insulin infusion in conscious, undisturbed rats, provided direct evidence that this postprandial, sodium-conserving action of insulin was due to insulin acting directly on the kidney^22^.

Insulin has been reported to stimulate sodium transport at multiple sites along the nephron^3,5,7,15,16,18,38,39^, including activation of ENaC^15,16,18^. To date, however, normal postprandial changes in ENaC activity had not been examined. We proposed that some of the renal sodium retention in the postprandial period is a result of increased ENaC activity stimulated by insulin from glucose-induced hyperinsulinemia following a meal. We tested this hypothesis in the present study by measuring ENaC activity in collecting duct tubules isolated from Sprague Dawley rat kidneys who were either fasted or given a short-term high-carbohydrate gel stimulus to replicate the effect of a meal. We determined that 4 hours post meal, the glucose and insulin levels of the postprandial rats are elevated (Figs 1a and 2), while the plasma Na^+^ levels were significantly decreased (Fig. 1b). While the decrease in plasma Na^+^ is small (about 2 mM), it has been shown that even a 1 mM increase in plasma sodium can be associated with about a 2 mmHg increase in systolic blood pressure^40^. Additionally, we would have liked to examine Na^+^ and K^+^ urinary excretion values to further investigate any changes to K^+^ secretion by the renal outer medullary K^+^ channel which is coupled inversely to ENaC Na^+^ reabsorption, but within this time frame we were unable to collect sufficient urine to make these measurements.

It has also been proposed that high glucose may affect ENaC expression in human collecting duct cells^41^, but in our animal model of normal physiology it is impossible to dissociate changes to glucose and insulin levels. Beyond this, the glucose starvation required to perform a several hour glucose stimulation study in a cell culture model would indubitably affect total cellular metabolism which could have a myriad of effects, and could be working through as yet unknown secondary pathways to impact ENaC. Thus we focused on insulin signaling, as the pathways by which insulin impacts ENaC are well established^13,15,16,18,20,23,25,29^. We found that in our postprandial rats, the *P*_*o*_ of ENaC increases (Fig. 3) while there are no changes in ENaC subunit expression (Fig. 4). Lack of changes in ENaC expression and simultaneous elevation in *P*_*o*_ are consistent with previous work from our lab that demonstrated insulin-mediated ENaC activation was a result of *P*_*o*_ changes rather than channel number^15,16^.

Insulin signaling in the kidney is also believed to impact SGK1 activity^14,17,23^, as well as several other regulatory kinases including with-no-lysine4 (WNK4)^26^ and protein kinase B (also known as Akt)^29^. It is important to note; however, that these studies have been performed in a number of different models with different time points, experimental read-outs, and insulin concentrations. We therefore interrogated Nedd4-2 expression and phosphorylation state, as it is a classical downstream target of SGK1 that directly impacts ENaC surface channel number. We did not see any changes at the 4 hour time point (Fig. 5), but this does not exclude SGK1 or other kinases from important signaling in normal postprandial Na^+^ regulation. Likely, this evolutionary Na^+^ preservation mechanism has both acute, rapidly activating effects which may involve kinase activity as well as prolonged effects that taper off with decreasing serum insulin levels. Further investigation into additional time points within our experimental set up could help elucidate the time frames of different aspects of this pathway. For example, in a biphasic response, perhaps short term effects result in shifts in phosphorylation events that act within minutes to an hour involving the PI3K-SGK1 pathway; however, after a prolonged and slowly decreasing stimulus, changes such as a shift in ENaC trafficking result in channels with higher *P*_*o*_ remaining at the membrane for longer periods of time. The ENaC subunits are non-coordinately regulated, and fully cleaved more active channels are believed to have a shorter half-life at the plasma membrane^42–44^. Several researchers have ongoing projects to assess these trafficking changes at the cellular level^30,42–46^, but have not addressed them within the context of normal physiological insulin signaling. This shift in trafficking would not be easily detected in our current model, as our current traces are only stable for minutes whereas internalization occurs over a time frame of 5–20 min and single channel patch clamp does not cover the surface area required to observe these changes. To more fully understand this mechanism would require addition of multiple time points as well as challenging radioactive labeling or surface internalization experiments, or mounting of opened renal tubules into a monolayer that could measure macro-currents for a prolonged amount of time.

In addition to ENaC expression, we also determined the total protein expression of NCC, NKCC2, and αNKA in the postprandial period (Fig. 6). While we did not see any significant changes to protein expression, this does not exclude these sodium transporters from playing a role in postprandial distal Na^+^ reclamation.

Lastly, we assessed whether there were any changes to the RAAS signaling cascade following a meal, since both Ang II and aldosterone are potent up-regulators of ENaC activity, and thus could be involved in increasing the ENaC *P*_*o*_. We measured plasma levels of all angiotensin derivatives from both the classical (Ang I (1–10), Ang II (1–8), Ang III (2–8), Ang IV (3–8)) and alternative (Ang II (1–8), Ang 1–7, Ang 1–5) degradation pathways. We saw no significant changes in Ang derivative expression levels in either pathway in the postprandial rats (Fig. 7). We verified that there were no differences in circulating aldosterone levels (Fig. 8a), and while there was a trend towards elevation of aldosterone following postprandial treatment, the average concentration of aldosterone in these rats was still well below the half maximal activation concentration for aldosterone binding to the mineralocorticoid receptor. We also quantified the Ang II/Ang I ratios as a surrogate measurement for ACE activity as well as the sum of Ang I and Ang II as a surrogate for plasma renin activity. Again, we saw no significant changes between the fasted or postprandial rats (Fig. 8c,d). These lack of changes suggest RAAS signaling is not an intermediary regulatory agent of Na^+^ transport via ENaC 4 hours after a meal stimulus; however, additional time points leading up to this point would help solidify this assessment. Therefore, it is likely that insulin-mediated changes to Na^+^ reabsorption is an independent regulatory axis, which strengthens the idea that insulin regulation in the kidney is an evolutionary trait to prevent salt wasting following a meal.

While this study implicates ENaC as an important postprandial regulator of sodium excretion, the activity of additional sodium transporters including SGLT2 requires further investigation. Beyond this, we also have yet to consider the role of insulin stimulation on potassium secretion. Previous studies have shown that K^+^-selective conductance via the renal basolateral potassium K_ir_4.1/5.1 channel increases following insulin stimulation which causes hyperpolarization of the cell membrane, thereby facilitating the increase in ENaC activity and corresponding Na^+^ reabsorption^47^. Also of interest is the broader context of the significance of this variation in Na^+^ reabsorption over the course of a day, which would likely involve activation of this pathways roughly 3 times per day. While this preservation of Na^+^ via ENaC and the distal nephron is essential, it is crucial to remember that ENaC is responsible for fine-tuning final sodium excretion, and normally accounts for about 2–5% of the total Na^+^ reclaimed by the nephron, so these changes, while important, are still small in the larger context of total corporeal sodium. Our current study is only one small step toward understanding how routine fluctuations in circulating insulin affect normal renal physiology. Further research into the postprandial regulation of electrolyte excretion will help us better understand how pathological changes such as hyperinsulinemia in type 2 diabetes or hypoinsulinemia in type 1 diabetes affect our physiology and potentially give rise to new treatment options or better management of disease progression.

## Methods

### Animals

Male Sprague Dawley rats were purchased from Charles River Laboratories (Wilmington, MA). Animals were maintained in a standard 12/12 dark/light cycle with water and food provided *ad libitum*. For postprandial studies 9–10 week old rats were fasted for at least 12 hours overnight. The following day each rat was given 11 g of the veterinary supplement, Supplical (Butler Schein, Dublin, OH; cat#029908), which is a gel formulation that is primarily a carbohydrate mixture (malt and corn syrups) containing 0.8% protein. 4 hours after the supplement or continued fasting, rats were euthanized under isoflurane anesthesia. Kidneys perfused with phosphate buffered saline and blood samples from the descending aorta were collected and used in downstream procedures. Animal use and welfare adhered to the *NIH Guide for the Care and Use of Laboratory Animals* following a protocol reviewed and approved by the IACUC at the Medical College of Wisconsin.

### Electrolyte analysis and Insulin ELISA

Plasma electrolyte concentrations and blood glucose levels were determined by ABL800 FLEX blood gas analyzer (Radiometer America, Brea, CA) just prior to euthanasia. Plasma insulin levels were determined using a Rat Insulin ELISA kit (RayBiotech, Norcross, GA) at a 2x dilution according to manufacturer’s instructions.

### Western blotting

For Western blot analysis, kidney cortical lysates were prepared as previously described^17,48^. Kidney tissue samples weighing between 15–25 mg were pulse sonicated in Laemmli buffer with a protease inhibitor cocktail (Roche, Basel Switzerland) to a concentration of 20 mg/ml. The resulting supernatant was subjected to SDS-PAGE, transferred onto nitrocellulose membrane (Millipore, Bedford MA), probed with antibodies and subsequently visualized by enhanced chemiluminescence (Amersham Biosciences Inc, Piscataway, NJ). For Western blots, we used the following antibodies: α-, β-, γ-ENaC, and NKCC2 antibodies (1:1000, Stressmarq Biosciences Inc, Vancouver, cat# SPC-403D, SPC-404D, SPC-405D, and SPC-401D, respectively), NCC antibody (1:1000, kindly provided by Dr. David Ellison, Oregon Health and Sciences University), α-NKA antibody (1:2000, Santa Cruz Biotech cat# sc-365091), and Nedd4-2 and pNedd4-2 (1:1000, Cell Signaling cat# 4013 S and 8063S, respectively). Western blot densitometry quantification was determined using Image Lab software (BioRad, Hercules, CA) and normalized to actin loading controls.

### Patch-clamp analysis

Patch clamp electrophysiology was used to assess ENaC activity in isolated, split-open cortical collecting duct (CCD) tubules. CCDs were isolated as described previously^48,49^. Briefly, kidneys were cut into thin slices (<1 mm) and then placed into an ice-cold physiologic saline solution (pH 7.4). CCD regions were mechanically isolated from these slices under a stereomicroscope by micro-dissection with forceps. The tubules were split open with sharpened micropipettes controlled with micromanipulators to gain access to the apical membrane. Single-channel recordings were acquired with Axopatch 200B amplifier (Mol. Devices, Sunnyvale, CA, USA) interfaced via a Digidata 1440A to a PC running the pClamp 10.2 suite software (Mol. Devices) and subsequently analyzed with Clampex 10.2. Currents were filtered with an 8-pole, low pass Bessel filter LPF-8 (Warner Inst., Hamden, CT) at 0.3 kHz. A typical bath solution was used (in mM): 150 NaCl, 1 CaCl_2_, 2 MgCl_2_, 10 HEPES (pH 7.4). The pipette solution for the cell-attached configuration was (in mM): 140 LiCl, 2 MgCl_2_ and 10 HEPES (pH 7.4). *NP*_*o*_, the product of the number of channels and the open probability (*P*_*o*_), was used to measure the channel activity within a patch. When multiple channel events were observed in a patch, the total number of functional channels (*N*) in the patch was determined. Event probability histograms were generated from computer determined single-channel event detection software in Clampfit10.2 and graphed in Origin9.0 (OriginLab, Northhampton, MA).

### Renin-angiotensin-aldosterone analysis

The characterization of the renin-angiotensin system components was performed by quantification of the steady-state angiotensin peptide and aldosterone levels in equilibrated heparin plasma samples by Attoquant Diagnostics (Vienna, Austria). Briefly, angiotensin peptide levels were measured following 30 min of equilibration in conditioned lithium-heparin plasma at 37 °C and subsequent stabilization of equilibrium peptide levels. Stable isotope-labeled internal standards for each angiotensin metabolite (Ang I, Ang II, Ang-(1–7), Ang-(1–5), Ang-(2–8), Ang-(3–8)) as well as the deuterated internal standard for aldosterone (aldosterone D4) were added to stabilized plasma samples at a concentration of 200 pg/ml and subjected to liquid chromatography tandem-mass spectrometry (LC-MS/MS)-based angiotensin and steroid quantification by Attoquant Diagnostics as described previously^50^. As previously reported, using protease inhibitor-stabilized and equilibrated samples for angiotensin peptide quantification resulted in similar qualitative outcomes^51^.

### Statistics

Results are presented as mean ± SEM. For analysis of data, equal variance and normality (Shapiro-Wilk) tests were performed followed by unpaired student t-tests to determined significance, and p-values of less than 0.05 are considered significant.

## Supplementary information

Supplementary_Uncropped blots
