# Supplementary material for: Postprandial Effects on ENaC-Mediated Sodium Absorption
Source: Sci Rep. 2019 Mar 12;9:4296. doi: 10.1038/s41598-019-40639-x (PMC6414683; doi:10.1038/s41598-019-40639-x)
Supplement: Supplementary file 1 — Supplementary_Uncropped blots [file 41598_2019_40639_MOESM1_ESM.pdf]

POSTPRANDIAL EFFECTS ON ENAC-MEDIATED SODIUM ABSORPTION

Gregory Blass[1,#,*], Christine A. Klemens[1,*], Michael W. Brands[2], Oleg Palygin[1], Alexander Staruschenko[1*]

[1]Department of Physiology, Medical College of Wisconsin, Milwaukee, WI 53226;
[2]Department of Physiology, Medical College of Georgia, Augusta University, Augusta, GA 30901

Running title: Postprandial effects on ENaC

[#]Present address: Western Kentucky University, Bowling Green, KY 42101

*Both authors contributed equally to this work

[*]**Corresponding author:** Alexander Staruschenko, PhD; Department of Physiology, Medical College of Wisconsin, 8701 Watertown Plank Road, Milwaukee, WI 53226, USA. Phone: (414) 955-8475; Fax: (414) 955-6546; E-mail: staruschenko@mcw.edu

aENaC

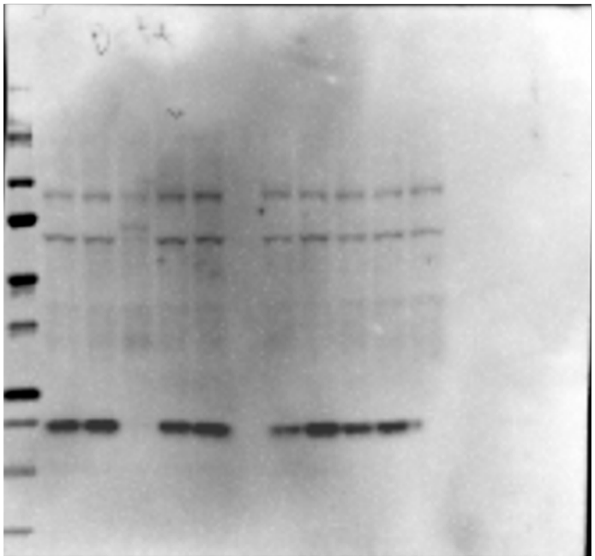

Actin

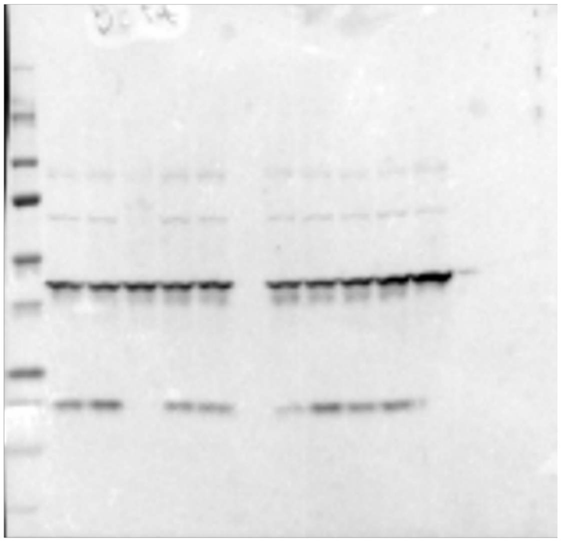

bENaC

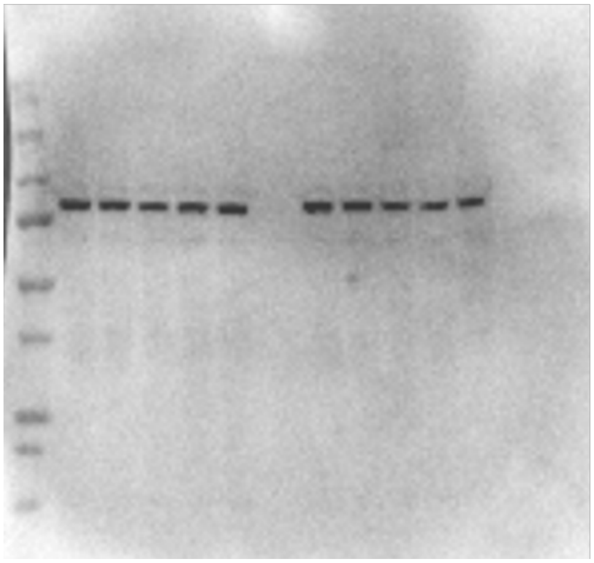

Actin

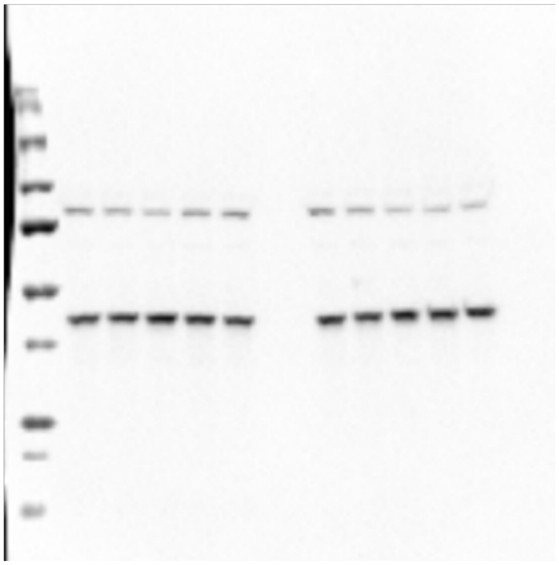

gENaC

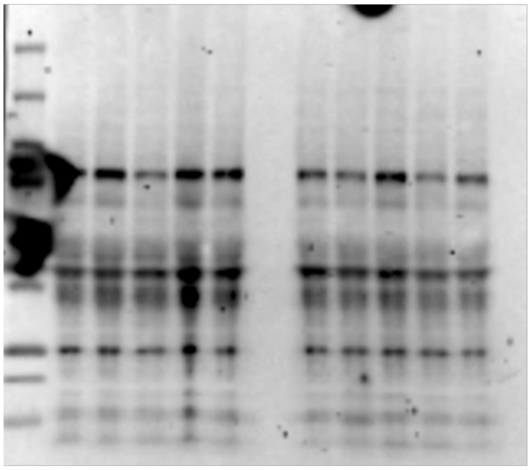

Actin

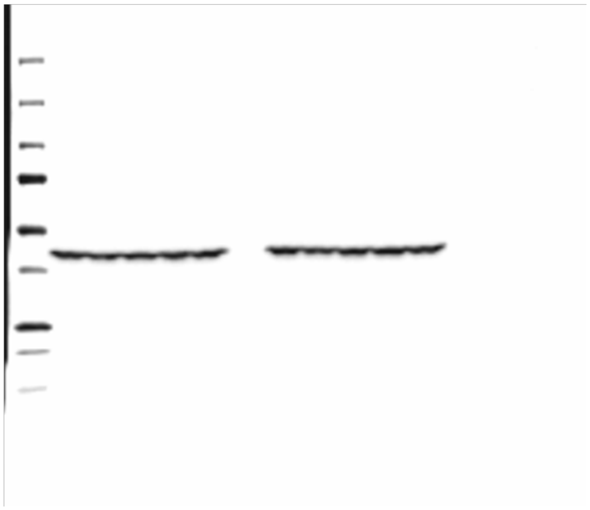

NCC

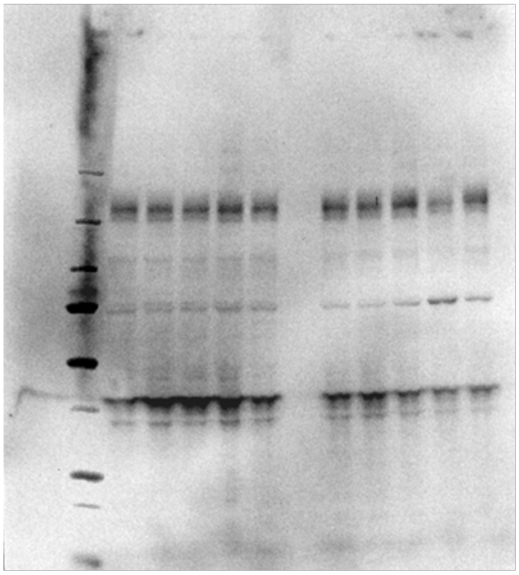

β-actin

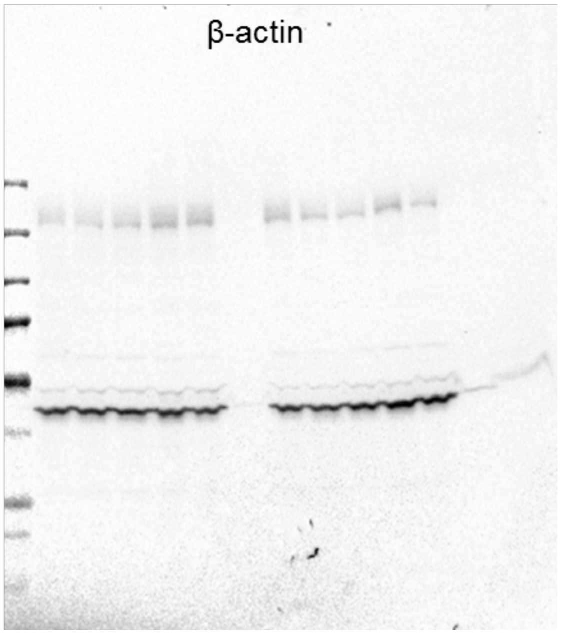

NKCC2

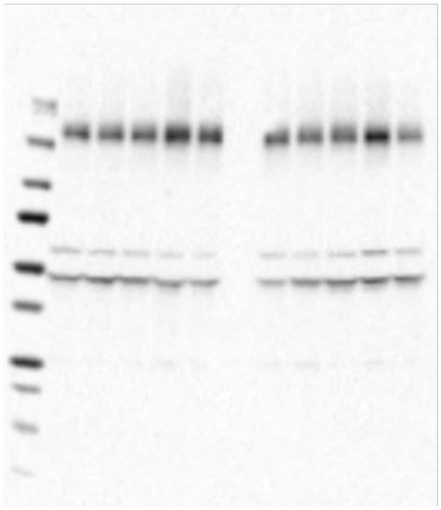

Actin

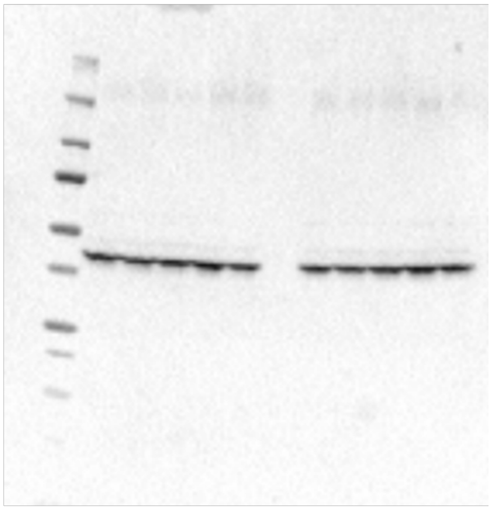

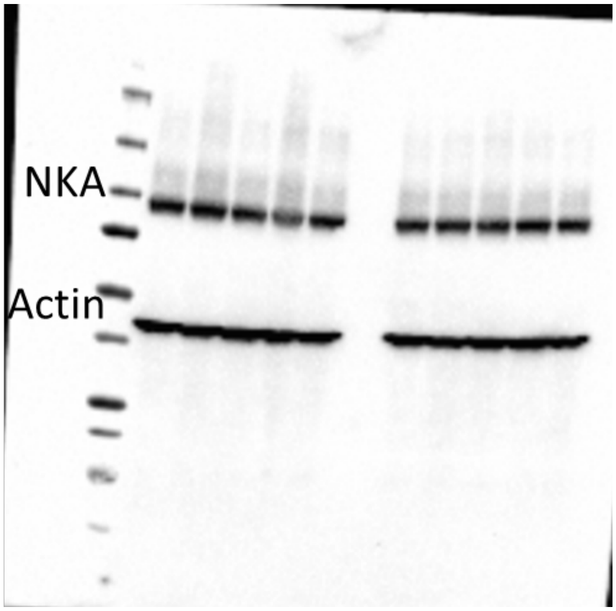

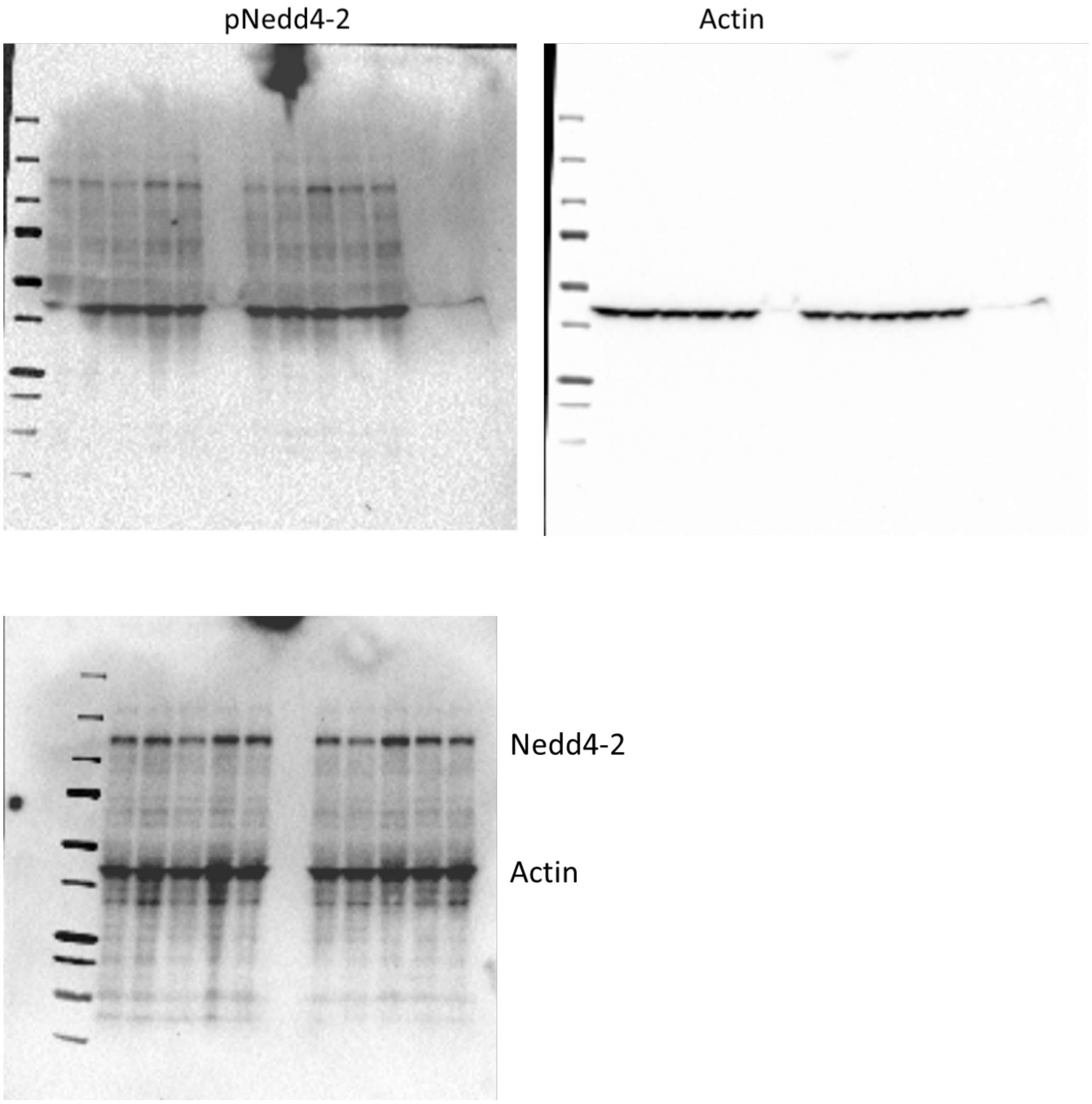

Supplementary Figure: Uncropped Western blots (Fig. 4, 5, 6).
